# Escitalopram co-prescription in anastrozole-treated breast cancer patients

**DOI:** 10.14744/nci.2022.48264

**Published:** 2022-07-19

**Authors:** Hazan Ozyurt, Sevgi Ozden, Cengiz Gemici, Esra Kucukibrahimoglu, Hatice Odabas, Neset Nesetoglu, Durisehvar Unal, Pinar Uler, Gokhan Yaprak, Huseyin Tepetam, Mahmut Gumus, Mehmet Zafer Goren

**Affiliations:** 1 Department of Radiation Oncology, Dr. Lutfi Kirdar Kartal Training and Research Hospital, Istanbul, Turkiye; 2 Department of Medical Pharmacology, Marmara University Faculty of Medicine, Istanbul, Turkiye; 3 Department of Medical Oncology, Dr. Lutfi Kirdar Kartal Training and Research Hospital, Istanbul, Turkiye; 4 Department of Analytical Chemistry, Istanbul University Faculty of Pharmacy, Istanbul, Turkiye; 5 Drug Application and Research Center, Istanbul University, Istanbul, Turkiye; 6 Department of Medical Oncology, Medeniyet University Faculty of Medicine, Istanbul, Turkiye

**Keywords:** Anastrozole, breast cancer, escitalopram, obesity, therapeutic drug monitoring

## Abstract

**OBJECTIVE:**

The purpose of the study was to evaluate the impact of escitalopram co-prescription on plasma anastrozole levels in post-menopausal breast cancer patients.

**METHODS:**

A total of 24 post-menopausal operated breast cancer patients co-prescribed with escitalopram and anastrozole were included. Blood samples were collected, before and 1-month after the onset of escitalopram to analyze plasma anastrozole and estradiol levels.

**RESULTS:**

No significant difference was noted in basal plasma anastrozole levels with respect to age, body mass index (BMI), tumor stage, previous antineoplastic treatments, concomitant medications, and serum estradiol levels. Overall, 17 patients completed the 1-month escitalopram treatment, while 7 patients discontinued escitalopram within the 1^st^ week of the treatment. Basal anastrozole levels of 24 patients were 26.1±2.4 ng/mL. Among 17 patients who continued 1-month escitalopram treatment was associated with significant increase in plasma anastrozole levels (24.5±2.3 ng/mL to 32.2±3.2 ng/mL, p<0.05). Notably, 1-month escitalopram use was associated with significant increase in plasma anastrozole levels only in the subgroup of obese (BMI >29 kg/m^2^) patients (23.1±2.8 to 35.9±4.7 ng/mL, p<0.01), while no such interaction was noted among non-obese patients. The estradiol levels of the patients were below ≤10 pg/mL in 75% of patients and no change occurred after escitalopram administration.

**CONCLUSION:**

Escitalopram co-prescription resulted in significant increase in plasma anastrozole levels without affecting the serum estradiol levels. Our findings emphasize the need for close monitoring in case of concomitant use of anastrozole and escitalopram, especially in obese patients and the potential role of therapeutic drug monitoring.

## Highlight key points

- In our cohort of post-menopausal breast cancer patients under anastrozole treatment, remarkable inter-individual variability in plasma anastrozole levels was revealed.
- No influence of age, BMI, tumor stage, previous anti-neoplastic treatment, concomitant medication on plasma anastrozole levels was detected.
- Escitalopram co-prescription resulted in significant increase in plasma anastrozole levels.
- In obese patients, escitalopram co-prescription was associated with more prominent increase in plasma anastrozole levels than non-obese patients.
- The measurement of anastrozole as a candidate for researches to identify variation in drug metabolism and emphasize the potential role of therapeutic drug monitoring in breast cancer patients, especially in obese patients.

Estrogen receptor (ER)-positive invasive type of breast cancer is common in postmenopausal women and these patients are therefore likely to obtain potential benefit from adjuvant endocrine therapy applied through tamoxifen or aromatase inhibitors (AIs) Third-generation AIs include exemestane, letrozole, and anastrozole [1, 2].

AIs or tamoxifen are considered acceptable as a standard care for the treatment of postmenopausal women with ER-positive invasive breast cancer [1]. AIs have increasingly become the preferred therapeutic option in postmenopausal breast cancer patients, given their efficacy superior to tamoxifen in prolonging disease-free survival and time to recurrence as well as in reducing the incidence of contralateral breast cancer and distant metastases [3–5].

Almost half of cancer patients are considered to suffer a co-morbid psychiatric or psychological disorder [6] while the prevalence of depression in women with breast cancer is estimated to be up to 27% [7]. Awareness and management of co-morbid depression are important among cancer patients since it has been associated with disturbed quality of life, physical deterioration, poor disease outcomes, and distressing symptoms that may persist years beyond the completion of antineoplastic treatment [7–11].

However, concomitant use of antidepressants and antineoplastic agents is a critical issue in terms of potential drug–drug interactions since both drugs have narrow therapeutic indices and metabolized mostly by CYP 450 enzymes indicating a likelihood of alteration in plasma drug concentrations and thus sub-therapeutic or toxic effects [12].

Impact of selective serotonin reuptake inhibitor (SSRI) antidepressants on CYP2D6-dependent drug metabolism may be important due to their widespread use, while their ability to inhibit the CYP2D6 enzyme is not uniform and ranges from strong inhibition (paroxetine and fluoxetine) to weak inhibition (citalopram and escitalopram) [12, 13].

The potential impact of CYP2D6 inhibition by SSRIs on clinical outcome among breast cancer patients under tamoxifen therapy become the most widely studied drug–drug interaction in breast cancer patients with co-morbid depression [13, 14]. However, debate continues on the adverse clinical outcomes associated with the use of CYP2D6 inhibitor antidepressants in breast cancer patients receiving tamoxifen [11].

Anastrozole is predominantly oxidized through phase 1 hydroxylation by CYP3A4 and less commonly by CYP 3A5, 2C8, 1A2, and 2C9 in the liver [1, 15]. The enzymes UGT1A4, UGT2B7, and UGT1A3 have also been suggested to participate in anastrozole metabolism [15]. CYP2C19, CYP3A4, and CYP2D6 are involved in the bio-transformation of the escitalopram with the ratios of 37%, 35%, and 28%, respectively [16]. However, escitalopram shows weak or negligible inhibition of CYP system including CYP 2D6 as well as CYP 1A2, 2C9, 2C19, and 3A4, suggesting a favorable pharmacokinetic profile with low potential for drug–drug interactions and thus clinical utility in a wide range of patients [17].

To our knowledge, no data are available on the drug–drug interactions for escitalopram and anastrozole in breast cancer patients. The present study was therefore designed to evaluate the impact of escitalopram co-prescription on plasma anastrozole levels in post-menopausal breast cancer patients treated with anastrozole.

## MATERIALS AND METHODS

### Study Population

A total of 24 operated post-menopausal breast cancer patients who were co-prescribed with escitalopram while taking anastrozole for at least 1 month were included in this study. The inclusion criteria were being on anastrozole treatment (1 mg/day) for at least 1 month (30±2 days), being younger than age of 75, having no implementation of radiotherapy or chemotherapy in the past 3 months before the study enrollment and having normal renal and hepatic functions.

Written informed consent was obtained from each subject following a detailed explanation of the objectives and protocol of the study which was conducted in accordance with the ethical principles stated in the “Declaration of Helsinki” and approved by the Dr. Lutfi Kirdar Kartal Training and Research Hospital (B104İSM4340029/1009/105, 25.12.2012).

### Assessments

Data on age, body mass index (BMI; kg/m^2^), stage of tumor, previous anti-neoplastic treatments, and concomitant medications were collected in each patient at study enrollment. Blood samples were collected twice, before and 1-month after the onset of escitalopram treatment to analyze change in plasma anastrozole as well as estradiol levels under escitalopram treatment. Anastrozole levels were also analyzed with respect to baseline characteristics including age (≤57 years vs. >57 years), BMI (≤29.9 kg/m^2^ vs. >29.9 kg/m^2^), radiotherapy (left vs. right breast, the side indicates the affected organs, none), chemotherapy (applied vs. none), tamoxifen (applied vs. none), tumor stage, estradiol levels (≤10 pg/mL vs. >10 pg/mL), concomitant medications (present vs. none) and in patients discontinued versus continued 1-month escitalopram treatment.

### Anastrozole and Escitalopram Dosage

Patients were on anastrozole (1 mg/day) for at least 1 month (30±2 days), while escitalopram was initiated with a dose of 10 mg/day for the first 7 days and then the dose was gradually elevated to 20 mg/day to enable tolerance.

### Blood Biochemistry Analysis

**Figure 1:**
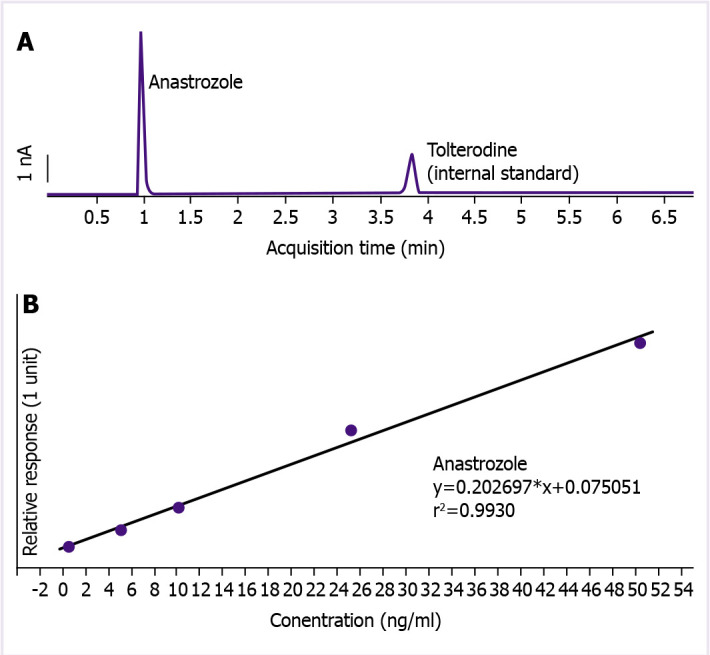
A representative chromatogram of elution of external standard injection into the HPLC system where tolterodine was used as an internal standard **(A)**, the regression analysis yielded an r^2^ of 0.9930 **(B)**.

Plasma anastrozole levels were measured twice, once before and once 1 month (30±2 days) after the onset of escitalopram by high-performance liquid chromatography (HPLC). The blood samples were centrifuged at 4500 g for 5 min within the 1 hour following the sampling and stored at -80°C until analysis. The plasma samples were extracted by using diethyl ether:dichloromethane (80:20, v:v) and high-performance liquid chromatography analyses were performed in Agilent Triple Quadrupole Mass Spectrometry system. A reverse phase C18 column (poroshell SB-C18, 2.7µm, 3.0 × 100 mm) was used within the system. The mobile phase was comprised of 5 mM ammonium acetate and acetonitrile at a ratio of 15:85 (v:v). Tolterodine was used as an internal standard. The external standard calibration interval was 0.5–50 ng/mL (r^2^: 0.9930). Before analysis, 5 mL venous blood samples were collected into Na-EDTA-containing glass tubes and the blood samples were centrifuged at 4500 g for 5 min within an hour following the sampling and stored at -80°C until analysis. A representative chromatogram is shown in Figure 1.

The time between the last dose of anastrozole and blood sampling was recorded. As there was a variation between the last anastrozole dose and the blood sampling time, the maximum plasma concentrations (C_max_) were extrapolated by using the following pharmacokinetic formula:

LogC=LogC_max_-k.t_1/2_; where k refers to elimination rate constant and t is sampling time as described previously by Shavi et al. [18].

### Statistical Analysis

Statistical analysis was made using GraphPad Prism version 5.00 for Windows (GraphPad Software, San Diego California, USA). Distribution of anastrozole levels (basal, before, and after escitalopram treatment) for normality analyzed by Shapiro–Wilk test. Data were expressed as “mean±standard error (SEM),” minimum-maximum, and percent (%) where appropriate. P<0.05 was considered statistically significant. The mean levels of anastrozole according to age, BMI, and previous treatments were compared by using unpaired Student’s t-test. Data were expressed as “mean±standard error (SEM),” minimum–maximum, and percent (%) where appropriate. P<0.05 was considered statistically significant.

## RESULTS

### Baseline Characteristics and Plasma Anastrozole Levels in the Overall Study Population (n=24)

Baseline characteristics of the patients are shown in Table 1. Overall 54.1% of patients aged ≤57 years, 62.5% had BMI of >29.9 kg/m^2^, and 79.1% had stage I to IIB disease. Previous antineoplastic treatments included adjuvant radiotherapy, chemotherapy, and tamoxifen in 87.5%, 79.1%, and 37.5% of patients and median time between completion of previous antineoplastic treatments and study enrollment was 3 years, 3.5 years, and 4 years for radiotherapy, chemotherapy, and tamoxifen, respectively. In 17 patients who continued to escitalopram treatment, 8 (47.1%) were receiving medications (i.e., anti-thyroid medications, vitamins) concomitant to anastrozole therapy (Table 2).

**Table 1:** Baseline characteristics and plasma anastrozole levels (n=24)

Characteristics	%	Anastrozole (ng/mL; mean±SEM)
Age (years)		
Median (min–max)	57.0 (41.0–73.0)	26.1±2.4 (10.3–52.3)
≤57	54.2	24.5±3.1
>57	45.8	28.1±3.6
BMI (kg/m^2^)		
Median (min–max)	30.4 (17.4–39)	
≤29.9	37.5	25.4±3.8
>29.9	62.5	22.9±2.8
Tumor stage*		
I	25	25±3.3
IIA	37	26.9±3.8
IIB	17	21.9±3.9
III	17	25.6±7.4
IV	4	52.2
Previous radiotherapy		
Left breast	20.8	30.7±6.4
Right breast	66.7	24.8±2.7
None	12.5	26.3±7.1
Previous chemotherapy		
Applied	79	25.3±2.8
None	21	25.7±5.2
Previous tamoxifen		
Yes	37.5	25.2±2.9
No	62.5	27.7±4.3
Concomitant medication		
Yes	62.5	25.9±3.9
No	37.5	25.8±2.1
Estradiol levels		
≤10 pg/mL	75	25.4±2.6
>10 pg/mL	25	28.5±6
Escitalopram		
Discontinued	29.2	31.0±5.7
Continued	70.8	24.2±2.3

* : n; SEM: Mean±standard error; BMI: Body mass index; Min: Minimum; Max: Maximum.

**Table 2:** Plasma anastrozole levels among patients continued to escitalopram treatment (n=17)

	Anastrozole (ng/mL)	
	Pre-escitalopram	Post-escitalopram
Overall (n=17)	24.5±2.3	32.2±3.2*
BMI		
>29 kg/m^2^ (n=10)	23±2.8	35.9±4.7**
≤29 kg/m^2^ (n=7)	26.3±4.9	26.1±7.1
Concomitant medication		
No (n=9)	25.8±2.1	32.1±5.1
Yes (n=8)	21.4±3.7	33.5±4.1
Levothyroxine (n=3)	22.4±11.0	34.6±15.0
Metoprolol (n=1)	17.8	40.4
Glucosamine (n=1)	18.9	27.9
Calcium+ vit D3 (n=1)	13.5	40.0
Risedronic acid + calcium + vit D3 (n=1)	44.8	24.0
Multivitamin complex (n=1)	15.4	31.9

BMI: Body mass index; Mean±SEM; *: P<0.05, Paired t-test; **: P<0.01, Paired t-test.

Serum estradiol levels were >10 pg/mL in 25% of patients. Overall, 17 (70.8%) patients completed the 1-month escitalopram treatment, while 7 (29.2%) patients discontinued escitalopram within the 1^st^ week of the treatment (Table 1).

Basal anastrozole levels of 24 patients were 26.1±2.4 (10.3–52.3) ng/mL. No significant difference was noted in basal plasma anastrozole levels with respect to age (≤57 years vs. >57 years), BMI (≤29.9 kg/m^2^ vs. >29.9 kg/m^2^), tumor stage, previous radiotherapy (left vs. right breast vs. none), chemotherapy (applied vs. none), tamoxifen (applied vs. none), concomitant medications (present vs. none), and serum estradiol levels (≤10 pg/mL vs. >10 pg/mL) (Table 1).

**Figure 2:**
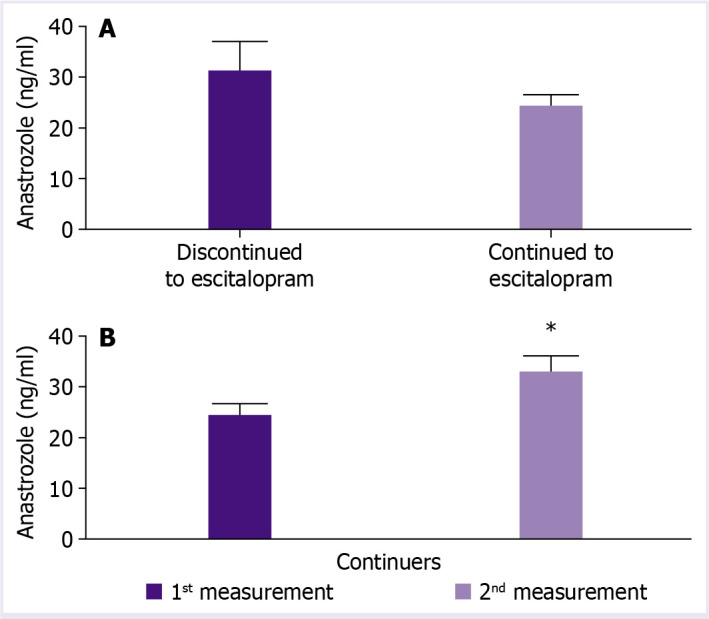
**(A)** Basal plasma anastrozole levels in discontinuers (n=7) vs. continuers (n=17) of 1-month escitalopram treatment; **(B)** anastrozole levels before (^1s^t measurement) and after (2^nd^ measurement) escitalopram treatment among continuers (n=17), *P<0.05; paired t-test.

Discontinuers of 1-month escitalopram treatment had higher basal plasma anastrozole levels than continuers (31±5.7 ng/mL vs. 24.5±2.3 ng/mL, Table 1, Fig. 2A).

### Plasma Anastrozole Concentrations According to Escitalopram among Continuers (n=17)

**Figure 3:**
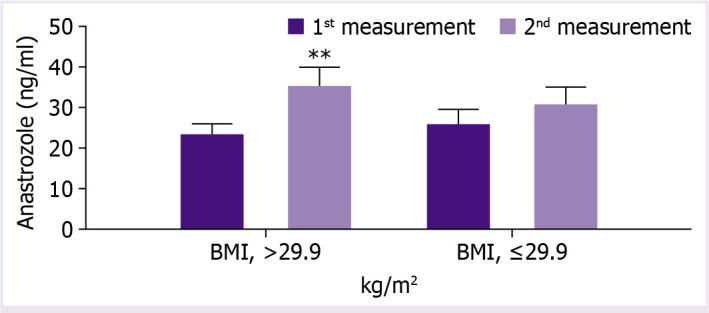
Plasma anastrozole levels compared to before (1^st^ measurement) and after (2^nd^ measurement) escitalopram treatment among continuers with BMI >29.9 kg/m^2^ (n=10) and among continuers with BMI ≤29.9 kg/m^2^ (n=7), **P<0.01; Paired t-test.

Among 17 patients who continued 1-month escitalopram treatment, the treatment was associated with significant increase in plasma anastrozole levels (from baseline 24.5±2.3 ng/mL to 32.2±3.2 ng/mL, p<0.05) (Table 2 and Fig. 2B). Increase in plasma anastrozole levels after escitalopram use was evident only in subgroup of obese (BMI >29 kg/m^2^) patients (from 23.1±2.8 to 35.9±4.7 ng/mL, p<0.01), while not in non-obese patients (Table 2 and Fig. 3).

### Serum Estradiol Concentrations, Liver and Kidney Functions among Continuers (n=17)

The estradiol levels of the patients were ≤10 pg/mL in 75% of patients and no change occurred after escitalopram administration. No significant change was observed in the liver and kidney functions of any of the patients after escitalopram administration.

## DISCUSSION

We have shown that escitalopram co-prescription with anastrozole in post-menopausal operated breast cancer patients was associated with a significant increase in plasma anastrozole levels, particularly among obese patients. No significant difference was shown in basal plasma anastrozole levels concerning age, BMI, tumor stage, previous antineoplastic treatment, concomitant medication, and serum estradiol levels. Our findings indicate the higher likelihood of escitalopram discontinuation in patients with higher basal plasma anastrozole levels and a higher likelihood of a post-escitalopram increase in plasma anastrozole levels in obese than non-obese continuers of antidepressant treatment.

Past studies revealed higher anastrozole and estradiol levels in obese than in normal weight patients [19, 20]. Several (patho) physiological alterations associated with obesity have been described in the literature, such as differences in metabolic or elimination processes, including phase I or phase II metabolisms, liver blood flow, glomerular filtration, and tubular processes, while their exact impact on specific drug metabolic and elimination pathways remains unknown. A trend toward lower CYP3A4 activity associated with obesity was also indicated for other major CYP3A4-cleared drugs [21]. Carbamazepine clearance in non-obese versus obese patients was reported to be marginally higher [22], while major weight loss, carbamazepine clearance in six obese patients was reported to be significantly increased [23]. Our findings related to increasing in anastrozole levels after escitalopram treatment in obese patients seem to indicate the likelihood of obesity to play a role in drug interaction, supporting the previously suggested role of obesity to alter the pharmacokinetics of some anticancer agents [24]. Furthermore, BMI was indicated to be a strong predictor of the increased plasma anastrozole concentration as well as higher estrogenic activity in postmenopausal breast cancer patients under anastrozole treatment [19]. Basal plasma anastrozole levels were not different in obese versus non-obese patients as well as in patients with serum estradiol levels ≤10 pg/mL versus >10 pg/mL before the onset of escitalopram in our cohort. Moreover, while basal plasma anastrozole levels did not differ concerning obesity or serum estradiol levels, escitalopram co-prescription resulted in increased plasma levels of anastrozole in obese patients without altering estradiol levels. Increased total-body aromatization in obese women is considered likely to be compensated by increased plasma concentrations of anastrozole [19, 25]. Moreover, an increase in anastrozole levels with escitalopram co-prescription might also be due to the redistribution of both drugs in obese patients.

A possible interaction of SSRIs with the estrogenic receptors leading to increased estradiol-induced activity in vitro has also been suggested [26]. However, based on the confirmed efficacy of escitalopram in post-menopausal women with breast cancer regardless of the demographics, form of cancer treatment, and hormone therapy, concomitant anti-estrogen therapy is not expected to limit the clinical effects of escitalopram [10].

Albeit extensive data are available regarding the effect of standard dosing of anastrozole on estradiol suppression, no definition exists for the appropriate range of plasma anastrozole concentrations to achieve therapeutic efficacy in breast cancer patients [19, 27].

Previous studies reported the anastrozole plasma steady-state concentrations as median 32.2 and mean 37.4 ng/mL (SD, 15.1–15.2) in breast cancer patients [19, 28–30]. The mean steady-state concentrations for anastrozole in our cohort seem to correspond to those values only after escitalopram co-prescription. Notably, plasma anastrozole levels among those who discontinued escitalopram within the 1^st^ week of treatment were already in the range of 10.33–52.3 ng/mL. Among continuers, post-escitalopram levels achieved 11.1–44.4 ng/mL and 9.1–57.0 ng/mL in our cohort before and after escitalopram, respectively. This seems consistent with the marked inter-individual variability of anastrozole concentrations (range from 0.0 to 98.8 ng/mL) reported in the past studies among breast cancer patients [19, 31].

Accordingly, given that it has no additional impact on estradiol levels, escitalopram co-prescription in our cohort seems to aid compensatory increase in anastrozole levels among obese breast cancer patients [19, 20], while this also indicates that co-prescription of anastrozole with escitalopram may necessitate close clinical monitoring.

In a past large-scale study on the metabolism and pharmacodynamics of anastrozole in breast cancer patients, substantial variability was noted in both drug metabolism and drug effect on circulating estrogens in postmenopausal patients [29]. Authors also indicated the potential contribution of marked variability in anastrozole metabolism to the drug efficacy and adverse events, necessitating the consideration of individualized therapy in anastrozole dosing in postmenopausal breast cancer patients [29].

Our findings support the consideration of anastrozole as a prime candidate for pharmacogenomic research to identify genetic variation in drug metabolism and emphasize the potential role of therapeutic drug monitoring to individualize and optimize adjuvant endocrine therapy in breast cancer patients [14, 29]. Our findings revealed no association of basal anastrozole levels with the potential confounding factors studied such as age, BMI, tumor stage, previous anti-neoplastic treatment, concomitant medication, and serum estradiol levels.

While an interaction between anastrozole and widely used chemotherapeutics in breast cancer patients such as cyclophosphamide, docetaxel, and paclitaxel is likely through inhibition of common metabolizer CYP3A4 [12, 31], our findings revealed no significant effects of previous chemotherapy on the basal level of anastrozole. The impact of radiation on drug pharmacokinetics through altering the protein and mRNA expression of drug-metabolizing enzymes such as cytochrome P450 and drug transporters has been suggested [32]. In an experimental study in rats, injection of neutron-activated UO2 particles (9.3 kBq) was reported to be associated with reduction in P450 enzyme-dependent xenobiotic metabolism by 30% on day 3 and by 40–70% on day 30, while the long-term effect has also been demonstrated that continues up to 1½ years leading to lung and liver xenobiotic activity at 30–60% and 60–75% levels, respectively [33]. Despite the possible interactions mentioned above, the median 3–4 years of gap between previous anti-neoplastic treatments and study enrollment in our cohort may explain why patients are not affected by previous treatments. Small sample size is obviously another factor that limited conduction and accuracy of subgroup analyses.

Patients with and without concomitant medications had higher anastrozole concentrations after escitalopram in our study, suggesting that concomitant medications did not change the effect of escitalopram on anastrozole level. Our findings support the need for larger scale studies addressing marked inter-individual variability in plasma anastrozole levels as well as importance of patient-specific pharmacokinetic and pharmacogenomic data to target anastrozole treatment [19, 29].

Owing to its low affinity for adrenergic, cholinergic, and histaminergic receptors, escitalopram is considered to have favorable tolerance with a relatively low incidence of anticholinergic, cardiovascular, and sedative side effects [34].

Nonetheless, one-third of patients discontinued escitalopram within the 1^st^ week of treatment in our cohort, despite the fact that escitalopram (5–20 mg/day) is associated with rapid amelioration of depression-related symptoms, within 1 week of therapy in breast cancer patients [11]. Similarly, the use of escitalopram 20 mg daily for 12 weeks to treat hot flushes among symptomatic postmenopausal breast cancer survivors revealed that 39% of patients never began therapy, while 14% of patients discontinued treatment due to side effects such as nausea and somnolence [35].

Anastrozole is predominantly oxidized through phase I hydroxylation by CYP3A4 and less commonly by CYP 3A5, 2C8, 1A2, and 2C9 in the liver [12, 15]. The enzymes UGT1A4, UGT2B7, and UGT1A3 [15] have also been suggested to participate in anastrozole metabolism. CYP2C19, CYP3A4, and CYP2D6 are involved in the bio-transformation of the escitalopram with the ratios of 37%, 35%, and 28%, respectively [16]. While pharmacokinetic mechanisms through inhibition of CYP 3A4 might indicate a potential interaction between anastrozole and escitalopram, no data are available in the literature regarding the potential drug–drug interactions between anastrozole and escitalopram in breast cancer patients as well as the pharmacokinetic mechanisms specific for escitalopram metabolism that could help to explain the mechanism of the interaction observed in our study.

Certain limitations to this study should be considered. First, relatively low sample size might prevent us to achieve the statistical significance concerning the impact of confounding factors on basal plasma anastrozole levels as well as to project our findings to the entire population. Second, the evaluation of estrogenic activity through serum estradiol rather than estrone sulfate levels is another limitation of our study. Estradiol levels are known to be low in post-menopausal women and measurements with crude methods are considered not effective for comparing the efficacy of AIs, while estrone sulfate levels are found within a measurable range and are considered to be more convenient in postmenopausal women [36]. Third, discontinuation of escitalopram in almost one-third of patients is another limitation which otherwise would extend the knowledge achieved in the current study. Nevertheless, despite these certain limitations, given the paucity of the solid information available on this subject, our findings represent a valuable contribution to the literature.

## Conclusion

In conclusion, our results in a cohort of postmenopausal breast cancer patients under anastrozole treatment revealed remarkable inter-individual variability in basal plasma anastrozole levels along with no influence of age, BMI, tumor stage, previous anti-neoplastic treatment, concomitant medication, and serum estradiol levels on plasma anastrozole levels. Escitalopram co-prescription resulted in a significant increase and marked inter-individual variability in plasma anastrozole levels, while no significant alteration was noted in serum estradiol levels. Our findings emphasize a possible need for close monitoring in case of concomitant use of anastrozole with escitalopram and the potential role of therapeutic drug monitoring to individualize and optimize adjuvant endocrine therapy among breast cancer patients. While escitalopram co-prescription might contribute to a compensatory increase in plasma anastrozole levels in obese patients, the clinical relevance of this finding warrants further investigation in terms of the association of anastrozole plasma levels with therapeutic efficacy, the role of confounding factors including the genetic variations on inter-individual variability in anastrozole metabolism and the specific pharmacokinetic information to reveal mechanisms underlying a potential drug–drug interaction.
